# Changes in breakfast and water consumption among adolescents in Canada: examining the impact of COVID-19 in worsening inequity

**DOI:** 10.1186/s40795-024-00831-3

**Published:** 2024-02-05

**Authors:** Markus J. Duncan, Emily Belita, Angelica Amores, Negin A. Riazi, Sarah Carsley, Leigh M. Vanderloo, Valerie Carson, Jean-Philippe Chaput, Guy Faulkner, Scott T. Leatherdale, Karen A. Patte

**Affiliations:** 1 Department of Health Sciences, Brock University, 1812 Sir Isaac Brock Way, St. Catharines, ON L2S 3A1 Canada; 2 School of Nursing, McMaster University, 1280 Main Street West, Hamilton, ON L8S 4K1 Canada; 3 School of Public Health Sciences, University of Waterloo, 200 University Ave West, Waterloo, ON N2L 3G1 Canada; 4 Public Health Ontario, 480 University Avenue, Suite 300, Toronto, ON M5G 1V2 Canada; 5 Dalla Lana School of Public Health, University of Toronto, 155 College St, Room 500, Toronto, ON M5T 3M7 Canada; 6 ParticipACTION, 77 Bloor St. W., Suite 1205, Toronto, ON M5S 1M2 Canada; 7 School of Occupational Therapy, Western University, 1201 Western Rd., London, ON N6G 1H1 Canada; 8 Faculty of Kinesiology, Sport, and Recreation, University of Alberta, 8840 114 St., Edmonton, AB T6G 2H9 Canada; 9 Healthy Active Living and Obesity Research Group, Children?s Hospital of Eastern Ontario Research Institute, 401 Smyth Road, Ottawa, ON K1H 8L1 Canada; 10 Department of Pediatrics, University of Ottawa, 75 Laurier Ave. East, Ottawa, ON K1N 6N5 Canada; 11 School of Kinesiology, University of British Columbia, Lower Mall Research Station, 2259 Lower Mall, Vancouver, BC V6T 1Z4 Canada

**Keywords:** Healthy eating, Breakfast, Water consumption, COVID-19, Equity, Longitudinal

## Abstract

**Background:**

To assess whether changes in breakfast and water consumption during the first full school year after the emergence of the COVID-19 pandemic varied based on sex/gender, race/ethnicity, and socioeconomic status among Canadian adolescents.

**Methods:**

Prospective annual survey data collected pre- (October 2019-March 2020) and post-COVID-19 onset (November 2020-June 2021) the Cannabis, Obesity, Mental health, Physical activity, Alcohol, Smoking, and Sedentary behaviour (COMPASS) study. The sample consisted of 8,128 students; mean (SD) age = 14.2 (1.3) years from a convenience sample of 41 Canadian secondary schools. At both timepoints self-reported breakfast and water consumption were dichotomized as daily or not. Multivariable logistic generalized estimating equations with school clustering were used to estimate differences in maintenance/adoption of daily consumption post-COVID-19 based on demographic factors, while controlling for pre-COVID-19 behaviour.

**Results:**

Adjusted odds ratios (AOR) with 95% confidence intervals are reported. Females (AOR = 0.71 [0.63, 0.79]) and lower socioeconomic status individuals (AOR_Lowest:Highest_=0.41 [0.16, 1.00]) were less likely to maintain/adopt daily breakfast consumption than male and higher socioeconomic status peers in the 2020–2021 school year. Black identifying individuals were less likely than all other racial/ethnic identities to maintain/adopt plain water consumption every day of the week (AOR = 0.33 [0.15, 0.75], *p* < 0.001). No significant interaction effects were detected.

**Conclusions:**

Results support the hypothesis that changes in nutritional behaviours were not equal across demographic groups. Female, lower socioeconomic status, and Black adolescents reported greater declines in healthy nutritional behaviours. Public health interventions to improve adherence to daily breakfast and water consumption should target these segments of the population.

**Trial Registration:**

Not a trial.

## Background

The emergence of the coronavirus disease 2019 (COVID-19) pandemic propelled the enactment of several public health measures, and a corresponding reduction in non-pandemic related public health programs. Across the globe, nationwide or city lockdowns, school closures, physical distancing policies, and isolation and quarantine parameters were commonplace [1]. While necessary to mitigate viral transmission, school closures prompted great concern as schools are a primary setting for public health interventions. Schools play a particularly critical role in cultivating the health of children and adolescents, especially those low-income households and underserved areas [2, 3]. Further, school closures and COVID-19 infections disproportionately impacted communities with higher densities of lower socioeconomic status (SES) and racialized populations, whereas rural regions experienced fewer lockdowns and case rates [4–6]. Thus, the pandemic and enacted public health measures potentially contributed to widening pre-existing inequities in youth heath. Further understanding is needed to inform efforts to mitigate sustained impacts.

Nutritional behaviours and dietary intake represent key modifiable predictors of various chronic diseases across the lifespan. Much work has been done to promote the adoption and maintenance of healthy dietary patterns in adolescence, as a period when healthy nutritional behaviours and diet quality tend to decline, and lifelong health habits often become established [7, 8]. Adverse dietary changes during the pandemic response appear likely [9], given the disruption of school nutrition programs [10], alongside heightened exposure to food insecurity [11–14], reduced daily structure and routine [15], and increased stress or boredom [15]. Though, some evidence points to positive shifts, including greater consumption of home-prepared food and family meals [9, 16]. International research exploring dietary consumption and nutritional behaviours among children and youth during the COVID-19 pandemic has consisted primarily of cross-sectional designs with reports of variable findings. A systematic review of studies examining the changes in eating habits of children and adolescents published between the emergence of COVID-19 in March 2020 up to April 2022 identified 39 studies, 34 of which used cross-sectional retrospective report [17]. Only four studies used a prospective longitudinal design, but none of these prospective studies measured breakfast or water consumption frequency [17]. Regular breakfast consumption is associated with numerous positive outcomes in adolescents such as better cognitive functioning and learning, diet quality, physical activity levels, metabolic health, and subjective well-being [18–26]. Canada’s food guide [27] recommends water as the “drink of choice” for daily consumption as an alternative to sugar sweetened and other calorically dense beverages. Of the five cross-sectional studies which examined breakfast consumption changes identified by the systematic review [17]: two reported an increase, two reported a decrease, and one reported no change in breakfast consumption [17]. Additionally, no studies from the COVID-19 era have reported youth water consumption patterns [17].

Some studies have discerned which groups may be at most risk of adverse effects based on factors such as gender, SES, and race/ethnicity. Youth nutritional behaviours during the pandemic differed across gender; for example, weekly fruit and vegetable intake and adherence to limitations on sugar-sweetened drinks were higher among girls than boys during confinement [28]. Increases in number of daily meals were higher among males compared to females [29]. The pandemic has also perpetuated food insecurity for families, particularly for those with children [11, 12]. Families experiencing food insecurity reported higher decreases in the total amount of food and fresh food in the home and increases in non-perishable, processed food [30]. Furthermore, disparities in relation to food security have been linked to race/ethnicity due to systemic racism and discrimination [31]. Racialized groups were at greater risk of being unable to afford food throughout the pandemic and self-reported less confidence in household food security compared to non-racialized groups [32]. Such differences in food access and security may result in different patterns of nutritional behaviour change.

Thus, longitudinal research on dietary intake and nutritional behaviours in adolescents during the pandemic remains limited and inconsistent; in particular, there is a lack of prospective evidence on healthy dietary patterns such as daily breakfast consumption [17]. Prospective and multi-level studies with pre-pandemic data are necessary to robustly examine within-individual changes and differential impacts in diverse populations and in varied contexts over time. Furthermore, much of the literature on the impact of COVID-19 on adolescent eating habits has focused on specific food group consumption trends, with limited information on water and breakfast consumption behaviours among youth using a prospective longitudinal approach.

The purpose of this study was to use data from a rolling cohort study of Canadian adolescents to assess whether changes in daily breakfast and water consumption during the first full school year after the emergence of COVID-19 varied based on sex/gender, race/ethnicity, and SES. Additionally, interaction effects were assessed to determine if intersections of demographic categories were associated with compounded inequality. Understanding the critical factors which influence healthy eating throughout the pandemic can provide better understanding of groups at increased risk for experiencing health disparities. It was anticipated that females, racial/ethnic minority individuals, and lower SES adolescents would not adopt or maintain daily breakfast and water consumption at the same rate as their male, white, and higher SES peers. These factors were expected to interact such that differences would be even greater among individuals who endorsed multiple demographic factor associated with worse outcomes.

## Methods

### Design & data collection

Data collected in year 8 (Y8, October 2019 - March 12, 2020) and year 9 (Y9, November 2020 - June 2021) of the Cannabis, Obesity, Mental health, Physical activity, Alcohol, Smoking, and Sedentary behaviour (COMPASS) study were used. The primary purpose of the COMPASS study is to track changes in multiple youth health behaviours and outcomes over time, in order to allow for the evaluation of natural experiments by simultaneously tracking changes in school programs, policies, or built environment [33]. Longitudinal linkage between annual administrations is achieved using a series of items to create a unique code for each respondent that ensures anonymity while allowing COMPASS researchers to link student’s data across years [34].

The COMPASS study collects data annually (2012–2027) from a rolling cohort of students attending a convenience sample of secondary schools across Canada. Participating schools were located in the provinces of Alberta, British Columbia, Ontario and Quebec. The COMPASS study has received ethical approval from the University of Waterloo Human Research Ethics Committee (ORE#30,118), Brock University Research Ethics Committee (REB#18–099), CIUSSS de la Capitale-Nationale–Université Laval (#MP-13-2017-1264), University of Alberta Research Ethics Office (Project #00040729), University of British Columbia (Reference #H17-00167), and participating school boards. Additional details regarding study methods can be found online (www.compass.uwaterloo.ca) or in print [33].

Student-level data were collected using the COMPASS student questionnaire [35]. Prior to school closures related to the early outbreak of COVID-19 in Canada (up to March 12th, 2020), questionnaires used a paper optical mark recognition survey designed to collect student-reported data from full school samples during one classroom period. Subsequently, student questionnaires were adapted to online administration [36]. Schools emailed students an initial survey link and one reminder; schools were encouraged to schedule class time for survey completion. All students attending participating schools were invited to participate using active-information passive-consent parental permission protocols, which are critical for collecting robust data among youth [37, 38]. Students could decline to participate at any time. Recruitment methods and annual school retention for this period is detailed further by Rezvani and colleagues [39]. In Y8, 29,770 students responded prior to school closures representing a 83.4% response rate based on school enrollment; in Y9, 53,469 students responded online representing a 58.0% response rate based on enrollment. Eleven schools dropped out between the Y8 and Y9 cycles [39]. In total, 8,274 responses were able to be linked longitudinally between Y8 and Y9. As communication with students was handled by their schools, rather than directly by COMPASS study personnel, individual reasons for non-participation in a given year are unavailable. Linked responses were excluded from these analyses if they did not report their year of education or listed their education year as “Other” (e.g., new immigrant classes available in Quebec).

### Measures

#### Healthy eating outcomes

During the transition to online survey delivery at Y9, several items related to nutritional behaviours (e.g., frequency of fruit/vegetable consumption) were altered compared to Y8 to make them more relevant to students learning from home [35, 36], thus limiting longitudinal analyses. This study focused on daily breakfast and plain water consumption, as response options for these two items remained comparable.

*Daily breakfast consumption* was self-reported with a single item “I eat breakfast every day” with a yes or no response option. No changes were made to this item between Y8 and Y9 administration.

*Plain water consumption* on the Y8 student questionnaire asked “in a usual week (7 days), on how many days do you drink each of the following? Water (plain)” with response options: “none”, “1 day”, “2–3 days”, “4–6 days”, “every day”. In the Y9 student questionnaire, this was replaced with a series of items asking participants to indicate on which days of the past week (Monday through Sunday) they consumed “plain” water. Initially, response options for the Y9 question were scored to match the ordered response options available in Y8, however, violation of the proportional odds assumption and low frequency of response categories less than 7 days/week led to poor performance of a proportional odds regression model. Ultimately, data from both Y8 and Y9 were dichotomized based on whether plain water had been consumed 7 days/week or less.

#### Demographic variables of interest

Individual demographic variables available for comparison included sex/gender, race, and SES. Sex/gender responses included: *male*, *female*, *I describe my gender in a different way*, and *I prefer not to say;* however, due to low frequencies, responses other than male and female were excluded. Race/ethnicity responses allowed individuals to select all that apply of: *Asian*, *Black*, *Latin American/Hispanic*, *White* or *Other*; individuals who identified multiple racial identities were recategorized as “Other”. SES was assessed with an item asking students to rate their perceived familial financial comfort relative to an average student in their class (*more comfortable, as comfortable*, or *less comfortable*).

#### Control variables

Models controlled for education year of the student, province, self-reported school mode (in person, online, or hybrid), regional urbanicity (rural, small urban, medium urban, or large urban) from Statistics Canada’s 2016 census data [40] matched based on school postal codes, and length of time between observation periods. Control variables were selected based on plausible factors in available data that may influence breakfast and water consumption habits [41] that could have been imbalanced between any of the demographic variables of interest (e.g., provincial differences in racial/ethnic composition). Additionally, length of time between observation periods was intended to adjust for the varying length between survey completion due to disruption in typical annual data collection patterns between measurements.

### Statistical analysis

As outcomes were dichotomized, an analysis of covariance approach was used to account for change by statistically controlling for prior behaviour [42] using a logistic generalized estimating equation (GEE) with an exchangeable correlation structure to account for school level clustering. As a result, effects can be interpreted as the likelihood of maintaining or adopting daily breakfast or water consumption at Y9. An adjusted odds ratios (AOR) above 1 means that a group was more likely to maintain/adopt positive nutritional behaviours relative to the reference. Analyses were conducted in R (v 4.3.1) [24]. Geepack [43]and emmeans [44] packages were used to conduct GEE and compare contrasts respectively. Categorical predictor variables were dummy coded while ordinal variables were orthogonal polynomial coded.

The first stage of analyses examined whether each variable of interest was associated to Y9 behaviour after controlling for Y8 behaviour, control variables, and other variables of interest. A single GEE was constructed conditioning Y9 behaviour on Y8 behaviour, control variables, and all variables of interest. The significance of the association between Y9 behaviour and variables of interest was tested using Type 3 analyses of effects. Statistically significant associations were assessed for specific group differences using pairwise post hoc comparisons.

In the second stage of analyses, a series of three GEE models tested the addition of an interaction term between all possible pairs of variables of interest to the model used to assess main effects. Interaction models were compared to the nested main effects GEE using a likelihood ratio test and the Quasi Information Criterion (QIC).

## Results

**Fig. 1:**
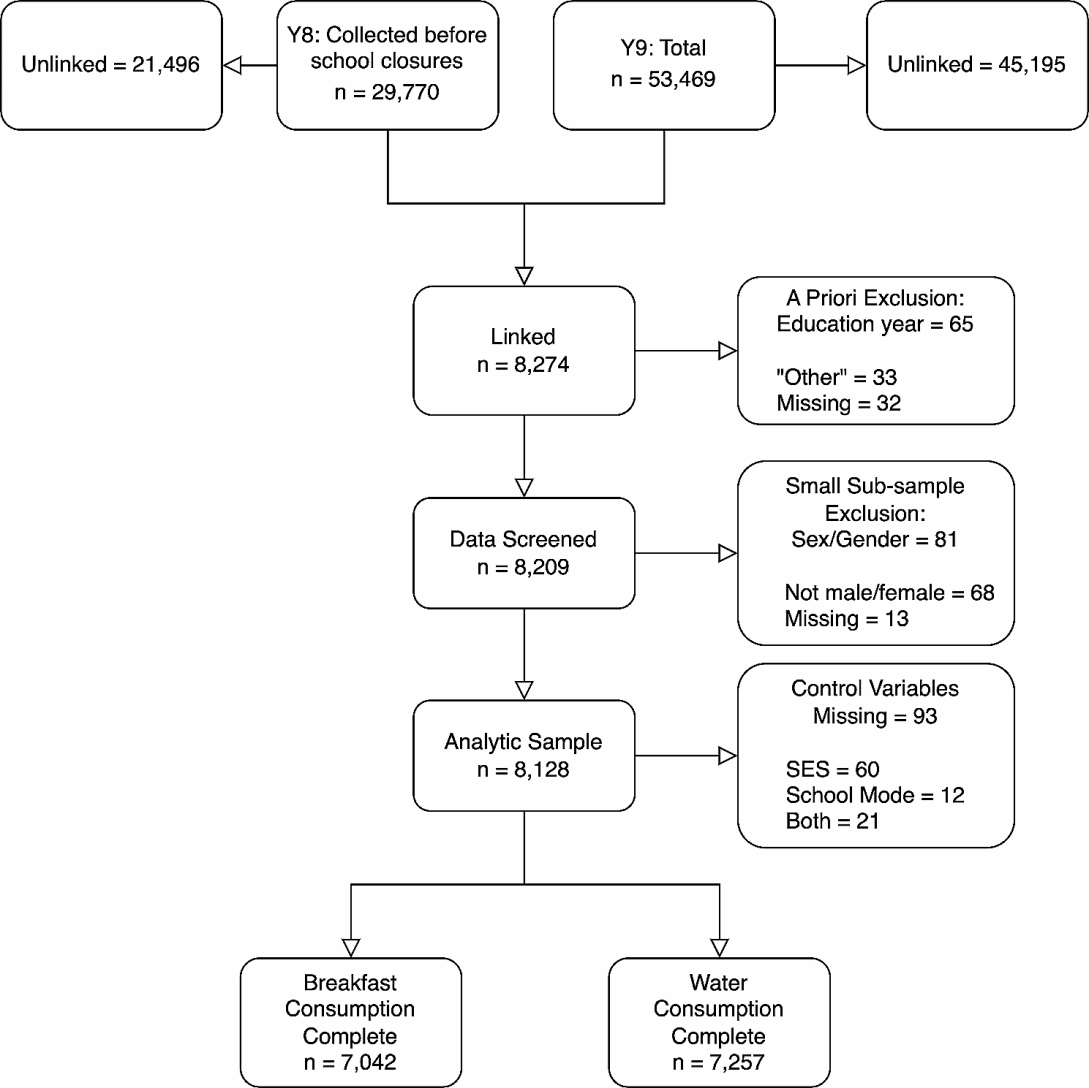
Flowchart of data used in analyses. *Note* Y8 = Year 8 (2019–2020). Y9 = Year 9 (2020–2021)

Available linked data consisted of 8,274 participants; individuals who did not report their year of education (*n* = 32) or listed their education year as “Other” (*n* = 33) were excluded. Additionally, due to low subsample size individuals did not report sex/gender (*n* = 13) or reported sex/gender other than male/female (*n* = 68) were excluded from analyses. The sample available for analysis consisted of 8,128 participants from 41 schools. Figure 1 depicts the link between the two cross-sectional data sources, data exclusion, and missingness in a flowchart. Two schools were located in Alberta, six in British Columbia, eighteen in Ontario, and seventeen in Quebec. The analyzed sample is described in Table 1. Mean age at Y8 was 14.2 (SD = 1.3) years.

**Table 1:** Sample characteristics

Variable	N / Mean	% / SD
**Province**		
Alberta	296	3.6%
British Columbia	1,075	13.2%
Ontario	2,545	31.3%
Quebec	4,212	51.8%
Y8 Education Year*		
*Secondaire 1*	1,271	15.6%
*Secondaire 2*	1,020	12.5%
9/*Secondaire 3*	2,481	30.5%
10/*Secondaire 4*	2,193	27.0%
11/*Secondaire 5*	1,110	13.7%
12	53	0.7%
**Sex/Gender**		
Female	4,604	56.6%
Male	3,524	43.4%
**Race/Ethnicity**		
Asian	694	8.5%
Black	108	1.3%
Latin	129	1.6%
White	6,218	76.5%
Other	979	12.0%
**Urbanicity**		
Large Urban	3,226	39.7%
Medium Urban	922	11.3%
Small Urban	1,445	17.8%
Rural	2,535	31.2%
**Relative SES**		
More Comfortable	2139	26.3%
As Comfortable	5,375	66.1%
Less Comfortable	533	6.6%
Missing	81	1.0%
**Y9 School Mode**		
In Person	2,119	26.1%
Mixed	2,516	31.0%
Online	3,460	42.6%
Missing	33	0.4%
**Y8 Breakfast Consumption**		
Every day	4,391	54.0%
Not every day	3,402	41.9%
Missing	335	4.1%
**Y9 Breakfast Consumption**		
Every day	4,032	49.6%
Not every day	3,629	44.6%
Missing	467	5.7%
**Y8 Water Consumption**		
7 days/week	6,591	81.1%
≤6 days/week	1,433	17.6%
Missing	104	1.3%
**Y9 Water Consumption**		
7 days/week	6,804	83.7%
≤6 days/week	832	10.2%
Missing	492	6.1%
**Days Between Observations**	458.8	(59.9)

*Note N* = 8,128 longitudinally linked students. *Secondary education in Quebec consists of Secondaire 1-5 at which point secondary education ends; Secondaire years are listed with the equivalent Grade systems used elsewhere in Canada where data were collected. Y8 = Year 8 (baseline; 2019-2020 school year; Pre-COVID-19 onset), Y9 = Year 9 (follow-up; 2020-2021 school year; Post-COVID-19 onset), SES = Socioeconomic status. SES scores range from 1 (lowest) to 5 (highest) based on composite of three items

Pairwise missing data allowed for analysis of *N* = 7,042 for breakfast consumption and *N* = 7,257 for water consumption. Unadjusted daily breakfast consumption from the pairwise complete cases was 57.0% in Y8 and 52.7% in Y9. Of individuals who were eating breakfast daily at Y8, 75.0% maintained daily breakfast consumption. Of individuals who were not eating breakfast daily at Y8, 23.2% became daily breakfast eaters. Unadjusted daily water consumption from the pairwise complete cases was 82.3% in Y8 and 89.5% in Y9. Unadjusted breakfast and water consumption stratified by variables of interest are provided in Table 2. Of individuals who were drinking water daily at Y8, 93.7% remained daily water consumers. Of individuals who were not drinking water daily at Y8, 70.0% became daily water consumers.

**Table 2:** Unadjusted proportion of participants consuming breakfast or plain water daily stratified by variables of interest

Group	Breakfast Consumption (*N* = 7,296)		Water Consumption (*N* = 7,488)		
	Y8 n (%)	Y9 n (%)	Y8 n (%)	Y9 n (%)	
**Sex**					
Female	2,143 (51.3)	1,969 (47.1)	3,570 (83.4)	3,808 (89.0)	
Male	1,997 (64.1)	1,870 (60.0)	2,585 (80.6)	2,870 (89.4)	
**Race/Ethnicity**					
Asian	307 (51.9)	302 (51.1)	539 (86.8)	570 (91.8)	
Black	37 (41.6)	35 (39.3)	71 (78.9)	69 (76.7)	
Latin	67 (58.8)	64 (56.1)	103 (85.1)	110 (90.9)	
White	3,338 (58.8)	3,041 (46.4)	4,738 (81.9)	5,172 (89.4)	
Other	391 (47.2)	397 (47.9)	704 (80.9)	757 (87.0)	
**Relative SES**					
More Comfortable	1,126 (58.4)	1,069 (55.4)	1637 (82.3)	1772 (89.0)	
As Comfortable	2,812 (57.6)	2,587 (53.0)	4118 (82.4)	4472 (89.5)	
Less Comfortable	202 (41.6)	183 (37.7)	400 (80.0)	434 (86.8)	

*Note* SES = Socioeconomic status. Data are pairwise complete used for modelling analyses. All percentages are the number of respondents indicating they engaged in daily breakfast and water consumption stratified by group

The GEE adjusted association between consuming breakfast daily at Y8 and Y9 consumption was AOR [95% Confidence Interval] = 9.36 [8.37, 10.49], meaning individuals who were daily breakfast eaters at Y8 were nearly 10 times as likely to continue eating breakfast daily as non-daily breakfast eaters at Y8 were to adopt daily breakfast consumption at Y9. The model adjusted association of daily water consumption at Y8 with Y9 consumption was AOR = 6.03 [5.14, 7.07], meaning individuals who had been daily water drinkers at Y8 were approximately 6 times as likely to continue drinking water daily as individuals who had been non-daily water consumers at Y8 were to adopt daily water consumption at Y9. Statistical results and effect size of post hoc pairwise comparisons between groups for significant variables of interest are summarized in Table 3.

**Table 3:** Pairwise group comparisons for significant omnibus differences from main effects models

Variable	Adjusted Odds Ratio [95% CI]	
Contrast	Breakfast Consumption (*N* = 7,296)	Water Consumption (*N* = 7,488)
**Sex**		
Female:Male	**0.71 [0.62, 0.81]**	0.87 [0.75, 1.01]
**Race/Ethnicity**		
Asian:Black	1.20 [0.64, 2.25]	**3.99 [1.48, 10.78]**
Asian:Latin	0.78 [0.47, 1.28]	1.31 [0.56, 3.07]
Asian:Other	0.91 [0.68, 1.23]	**1.65 [1.14, 2.40]**
Asian:White	0.92 [0.68, 1.24]	**1.50 [1.07, 2.10]**
Black:Latin	0.65 [0.31, 1.38]	0.33 [0.09, 1.20]
Black:Other	0.76 [0.41, 1.43]	0.41 [0.16, 1.09]
Black:White	0.77 [0.40, 1.47]	**0.38 [0.15, 0.92]**
Latin:Other	1.18 [0.75, 1.86]	1.26 [0.58, 2.74]
Latin:White	1.18 [0.72, 1.96]	1.15 [0.56, 2.32]
Other:White	1.00 [0.82, 1.23]	0.91 [0.67, 1.23]
**Relative SES**		
More:As Comfortable	1.08 [0.94, 1.25]	0.92 [0.74, 1.14]
More:Less Comfortable	**1.63 [1.19, 2.24]**	1.07 [0.76, 1.49]
As:Less Comfortable	**1.51 [1.10, 2.06]**	1.16 [0.86, 1.56]

*Note* CI = Tukey adjusted Confidence Interval, CIs that do not cross 1 are bolded. SES = Socioeconomic status; SES scores range from 1 (lowest) to 5 (highest) based on composite of three items. Adjusted odds ratio controlling for prior (Year 8) behaviour, sex/gender, race/ethnicity, SES score, urbanicity, education year, province, and length of time between observation periods

Daily breakfast consumption at Y9 varied based on sex/gender (χ^2^(1) = 27.4, *p* < 0.001) and SES (χ^2^(2) = 13.4, *p* = 0.001) when controlling for previous behaviour; no racial/ethnic differences were detected (χ^2^(4) = 2.9, *p* = 0.57). Females were less likely to maintain/adopt daily breakfast consumption compared to males AOR = 0.71 [0.62, 0.81]. There was a general trend where individuals with higher SES scores were more likely to maintain/adopt eating breakfast every day. Pairwise group contrasts are fully detailed in Table 3. Individuals who reported being less financially comfortable than classmates were significantly less likely to eat breakfast daily than those who said they were more or as comfortable as the average peer in their school.

Daily water consumption differed solely based on race/ethnicity (χ^2^(4) = 23.0, *p* < 0.001). Pairwise group contrasts are reported in Table 3. Black identifying individuals were significantly less likely to maintain/adopt daily water consumption than all other racial/ethnic groups. Additionally, Asian identifying individuals were significantly more likely to maintain/adopt daily water consumption compared to “Other” categorized individuals. Overall, sex/gender (χ^2^(1) = 3.5, *p* = 0.06) and SES (χ^2^(2) = 1.9, *p* = 0.38) differences in water consumptionwere non-significant.

Interaction models did not improve upon the respective nested main effects model; Table 4 summarizes the statistical results of model comparisons. Thus, no post hoc analyses were performed.

**Table 4:** Model comparisons of interaction models against the main effects model

		Breakfast Consumption (*N* = 7,042)					Water Consumption (*N* = 7,257)		
Interaction	*df*		*χ* ^*2*^	*p*	*QIC*	*χ* ^*2*^		*p*	QIC
Main Effect Model					8,032				4,608
Sex/Gender × Race/ethnicity	4		1.8	0.91	8,038	3.6		0.47	4,612
Race/Ethnicity × SES	8		8.9	0.35	8,042	375		1	8,550
Sex/Gender × SES	4		0.6	0.73	8,035	1.0		0.61	4,611

*Note* SES = Socioeconomic status. Results are for likelihood ratio tests comparing adding an interaction term to the main effects models used in Table 2, in addition to the listed variables of interest, models adjusted for prior (Year 8) behaviour, education year, province, regional urbanicity, and length of time between observation periods

## Discussion

In a cohort of Canadian secondary school students, we found evidence that changes in daily eating habits from before the COVID-19 pandemic response compared to the first full school year after the emergence of COVID-19 differed based on sex/gender, SES, and race/ethnicity. Specifically, females and lower SES adolescents were less likely to maintain/adopt daily breakfast consumption in the 2020–2021 school year than their male and higher SES peers; while Black identifying adolescents were less likely than students identifying as all other racial/ethnic identities to have maintained/adopted daily consumption of plain water. The lack of significant interactions suggests these inequities were not further compounded by intersections with the other individual level demographic factors.

United States data indicate that prior to COVID-19, Black children and adolescents tended to consume less water per day [45–47] and were less well hydrated than their white peers [48]. Similarly, we found that Black students were less likely than their peers to consume even a single serving of plain water each day when controlling for prior behaviour. Scant research has explored water consumption by racial and ethnic identity among Canadian adolescents. A pre-pandemic study of elementary school students (ages 8–14) found no difference in daily water consumption between Caucasian children and those of other ethnicities [49]. While the current study did not assess other types of beverage consumption, there is some previous evidence that Black adolescents may be disproportionately consuming sports drinks as a form of hydration [46], which are often sugar sweetened, and soft drinks [47]. These differences have been attributed to marketing of soft drinks and sports beverages targeted towards Black adolescents [50]. United States research has also found Black adolescents to be less likely than their white counterparts to have access to trusted, quality, tap water in schools [51, 52]. We found no evidence indicating differential changes in water consumption by sex/gender, or SES, contrary to pre-COVID-19 research that has generally found higher intake in girls and females than boys and males [49, 53] and in households with higher parental education [54]. The majority of research on child and adolescent water consumption has examined individual determinants; there remains a need to examine socioenvironmental and cross-level predictors that may contribute to disparities [41].

Only about half of adolescents reported eating breakfast every day, with a lower proportion reporting daily breakfast consumption in the post-COVID-19 onset year than prior to the pandemic. In a nationally-representative Canadian sample, 57% of boys and 48% of girls reported daily breakfast consumption in 2010, up from 55% to 45% in 2002, respectively [55]. Continued research is needed to monitor potential sustained and disparate impacts of the pandemic on regular breakfast consumption. Breakfast eating commonly declines over adolescence and nutritional behaviours in general become more irregular and exploratory, potentially due to age-related increases in autonomy over food choices and the growing influence of peers [55–57]. Regular breakfast consumption has been shown to have positive associations with adolescents’ cognitive functioning and learning, diet quality, physical activity levels, metabolic health, and psychosocial well-being [18–26]. Therefore, encouraging daily breakfast consumption has potential short and long-term benefits for adolescent health and well-being.

Consistent with the current results, pre-pandemic research has found higher rates of breakfast skipping among females and lower SES adolescents [55, 58, 59]. The most common reasons for skipping breakfast reported before the pandemic by Canadian adolescents include a lack of time and hunger in the morning and sleeping in [59–61]. These reasons may, in part, reflect their need for greater morning sleep, due to the misalignment between early school start times and delayed circadian cycles in adolescence. While school learning modes were adjusted for in this study, learning from home may have permitted more time for breakfast; however, evidence points towards shifts to later bedtimes and waketimes during lockdown [62]. Female adolescents are more likely than males to report skipping breakfast for these reasons, as well as feeling sick when they eat breakfast and trying to lose weight [59]. Despite evidence to the contrary [58], girls commonly believe that skipping breakfast is an effective means to control their weight [61, 63]. Evidence points to increased onset and exacerbation of disordered eating and poor body image among adolescents during the pandemic [64].

Our results for breakfast consumption by SES may partially reflect the heightened experiences of food insecurity during the pandemic [11–14] and disrupted access to school nutrition programs. School breakfast programs are intended to ensure all students have access to a nutritious meal and are targeted at students living in low income and food insecure households, but this was disrupted during acute phases of the COVID-19 pandemic. However, despite the need for school breakfast programs, participation rates are limited in secondary schools [65]. Even when school breakfast programs are provided, skipping breakfast and low nutritional quality foods in the morning were common among middle-grade US students regardless of household food security status [66]. Furthermore, a high proportion of youth that actively engage in school breakfast program still report skipping breakfast [65]. Canada remains the only high-income country without a national school food program; the delivery models of Canadian schools that do offer breakfast programs at no cost to students vary greatly, from grab-and-go baskets to sit-down meals [65, 67, 68]. Recommendations of program delivery include serving breakfast in classrooms, making programs available to everyone to discourage stigma, and “program champions” and student volunteers to encourage participation [65, 67].

A key strength of this study is the prospective design which reduces the risk of retrospective bias for pre-COVID-19 baseline data. The sample consists of a large sample of Canadian adolescents across four provinces, which is substantially larger than previous prospective studies examining the change of behaviours in children and adolescents during the pandemic [17]. The sample size allowed for even small differences between groups to be detected. Furthermore, the analyses considered possible interactions between variables to evaluate whether intersections between identity, economic status, and living environment may have contributed to compounding risk of poor eating habits; however, these interactions were not statistically significant. Based on a 2022 review of COVID-19 era literature [17], this is the only prospective study of adolescents to examine changes in either daily breakfast or water consumption and the only prospective Canadian data on nutritional behaviours in youth. Additionally, this study expands on early knowledge by examining a later phase of the pandemic after the initial crisis and acute interruptions to services, and thus may better represent stabilized changes in nutritional behaviour.

Despite the prospective, longitudinal nature of the data, one potential source of bias is that only 27.8% of the sample recruited at Y8 could be linked to data at Y9, which may have favoured response from participants who are generally conscientious, including about matters of health and health behaviour. Cross-sectionally the participation relative to enrollment dropped from 83.4% in Y8 to 58.0% in Y9. This decrease is unsurprising given that not all schools devoted time in the school day to participation and invitations to participate relied on e-mail solicitation rather than in-person recruitment. The shift to online surveys during COVID-19 may also have favoured individuals with more stable homelives who were thus able to devote time to completing the survey if no time was set aside by the school. Unlike a typical prospective cohort design the COMPASS study uses repeated cross-sectional data collection that is then linked longitudinally using a series of items. In addition to recruiting at the school-level rather than specific individuals, this provides students with additional anonymity when responding to health-related questions. However, with no direct communication to participants reasons for unsuccessful linkage are numerous and unclear.

Results of this study are likely reflective of the Canadian context, though larger subsample sizes of various demographics (e.g. Black and Latino students) is likely warranted to get a more accurate estimate of behaviour change in these populations. However, schools in Manitoba, Saskatchewan, Atlantic Canada and the northern territories did not participate which may reflect a variety of differences on cost of living, population/population density, and environment compared to Alberta, British Columbia, Ontario and Quebec. The sample size was not large enough to stratify to determine if the results of this analysis were similar across those provinces that were represented in the data or whether internal differences existed. Externally, the results are likely to be most applicable to high developed/high income countries with a modal white population.

A limitation of this study is that water and breakfast consumption were the only nutritional behaviours that could be assessed prospectively. When moving the COMPASS student questionnaire to online administration, several questions were updated; among those revised questions were fruit and vegetable consumption. While the water consumption item was revised from asking for the number of days in the past week (with response options on a 5 point Likert-like scale ranging from “none” to “every day”) to a question asking to indicate for specific days of the week when plain water was consumed (thus allowing future survey administrations to compare weekday and weekend consumption separately), both versions still specified “plain” water and response options could be scored on the same scale. Nevertheless, some of the changes (or lack thereof) in water consumption may be attributable to changes in the question format. Additionally, as the primary purpose was to identify population subgroups which experienced the greatest amount of change in daily behaviours during the first full school year after the emergence of COVID-19, we did not evaluate whether this rate of change is different than what occurs in a typical year. Furthermore, the data on breakfast consumption does not account for the composition of the meal, and thus we were not able to assess whether changes in breakfast consumption changed in terms of nutrients or food group components. Furthermore, we did not distinguish between students who may have not been able to consume breakfast due to financial reasons as opposed to personal choice or other reasons; such an analysis would have required substantially more complicated models which account for both breakfast consumption status and reasons at baseline and follow-up. Similarly, the data on water consumption did not account for the volume of plain water consumed and does not assess whether other non-sugar sweetened beverages such as tea or carbonated/seltzer water. Given the high rates of daily water consumption this may be of limited concern, however some individuals may be categorized as non-adherent despite consuming other non-sugar sweetened beverages for hydration. Additionally, there were no items assessing whether sugar sweetened beverages or other less healthful beverages were consumed even when water is reportedly drunk and the data could not distinguish between behaviour on school-days and non-school days. Ideally, both behavioural targets should be adhered to on all days, however such distinction would better clarify how to tackle issues of inequity in breakfast and water consumption. Finally, the modelling approach used where follow-up results are conditioned on baseline measures (often called an Analysis of Covariance approach) has been criticized as potentially introducing bias in observational studies [69, 70] with the advice that unadjusted change scores should be used in these circumstance [69, 70]. However, given the ordered nature of the data, applying this advice is problematic due to unequal spacing between arbitrarily coded values [71, 72]; as a result we opted to use the current approach to avoid violating the unequal spacing theory at the cost of potentially introducing bias.

## Conclusions

The evidence presented suggests that after the emergence of COVID-19, female adolescents and adolescents of low SES were less likely to adopt or maintain daily breakfast consumption the following school year. These groups of adolescents were already less likely to consume breakfast before the emergence of COVID-19 [55, 58, 59]. Similarly, Black adolescents were the only group to have lower rates of maintenance or adoption of daily water consumption; while pre-COVID-19 evidence found no difference by race among younger children in Canada [49], lower water consumption is a known concern among Black adolescents in United States [45–47]. Canadian public health stakeholders should take note that poor water consumption habits in Black adolescents may be an emerging or under researched health concern. Tailored interventions to change systematic barriers to these daily behaviours in these subpopulations continue to be warranted such as campaigns to promote water consumption, restrict food marketing to young people, and reduce potential barriers such as cost and quality of water in schools with large Black populations, school breakfast programs to mitigate income inequality, or education programs to dispel myths about skipping breakfast directed towards girls.

## Data Availability

The datasets used and analysed during the current study are available from the corresponding author with permission from the COMPASS study leadership on reasonable request.

## Abbreviations

COVID-19: Coronavirus Disease 2019
SES: socioeconomic status
Y8: year 8/baseline:October 2019 - March 12, 2020
Y9: year 9/follow-up:November 2020 - June 2021
COMPASS: Cannabis, Obesity, Mental health, Physical activity, Alcohol, Smoking, and Sedentary behaviour
GEE: Generalized Estimating Equation
QIC: Quasi Information Criterion
CI: Confidence Interval
